# Validation of a genome-wide polygenic score in improving fracture risk assessment beyond the FRAX tool in the Women’s Health Initiative study

**DOI:** 10.1371/journal.pone.0286689

**Published:** 2023-06-01

**Authors:** Xiangxue Xiao, Qing Wu

**Affiliations:** 1 Nevada Institute of Personalized Medicine, College of Science, University of Nevada, Las Vegas, Nevada, United States of America; 2 Department of Epidemiology and Biostatistics, School of Public Health, the University of Nevada Las Vegas, Las Vegas, Nevada, United States of America; 3 Department of Biomedical Informatics, College of Medicine, The Ohio State University, Columbus, OH, United States of America; Universitair Kinderziekenhuis Koningin Fabiola: Hopital Universitaire des Enfants Reine Fabiola, BELGIUM

## Abstract

**Background:**

Previous study has established two polygenic scores (PGSs) related to femoral neck bone mineral density (BMD) (*PGS*_*FNBMD*_*ldpred*_) and total body BMD (*PGS*_*TBBMD*_*ldpred*_) that are associated with fracture risk. However, these findings have not yet been externally validated in an independent cohort.

**Objectives:**

This study aimed to validate the predictive performance of the two established PGSs and to investigate whether adding PGSs to the Fracture Risk Assessment Tool (FRAX) improves the predictive ability of FRAX in identifying women at high risk of major osteoporotic fracture (MOF) and hip fractures (HF).

**Methods:**

The study used the Women’s Health Initiative (WHI) cohort of 9,000 postmenopausal women of European ancestry. Cox Proportional Hazard Models were used to assess the association between each PGS and MOF/HF risk. Four models were formulated to investigate the effect of adding PGSs to the FRAX risk factors: (1) Base model: FRAX risk factors; (2) Base model + *PGS*_*FNBMD*_*ldpred*_; (3) Base model + *PGS*_*TBBMD*_*ldpred*_; (4) Base model + metaPGS. The reclassification ability of models with PGS was further assessed using the Net Reclassification Improvement (NRI) and the Integrated discrimination improvement (IDI).

**Results:**

The study found that the PGSs were not significantly associated with MOF or HF after adjusting for FRAX risk factors. The FRAX base model showed moderate discrimination of MOF and HF, with a C-index of 0.623 (95% CI, 0.609 to 0.641) and 0.702 (95% CI, 0.609 to 0.718), respectively. Adding PGSs to the base FRAX model did not improve the ability to discriminate MOF or HF. Reclassification analysis showed that compared to the model without PGS, the model with *PGS*_*TBBMD*_*ldpred*_ (1.2%, p = 0.04) and metaPGS (1.7%, p = 0.05) improve the reclassification of HF, but not MOF.

**Conclusions:**

The findings suggested that incorporating genetic information into the FRAX tool has minimal improvement in predicting HF risk for elderly Caucasian women. These results highlight the need for further research to identify other factors that may contribute to fracture risk in elderly Caucasian women.

## Introduction

Osteoporosis is a common bone disease that increases predisposition to fractures [1]. Worldwide, osteoporotic fractures have become a critical public health issue with a high post-fracture disability and mortality rate, resulting in social and economic burdens [2]. Early detection of the high-risk population could lead to efficacious preventive and therapeutic interventions.

Prior studies have demonstrated the polygenic nature of fractures [3–6]. The predisposition to osteoporotic fracture is attributable to a complex interaction between genetic and non-genetic factors [7]. Many assessment tools have been developed to identify susceptible individuals with a higher propensity to get clinically relevant fractures that merit an intervention [8–10]. However, the personalized genetic predisposition of experiencing a fracture was not incorporated into any of those tools. The Fracture Risk Assessment Tool (FRAX) is the most widely used risk stratification tool in North America. FRAX was used to assess the 10-year probability of major osteoporotic fracture (MOF) and hip fracture (HF) on an individual level using 12 clinical risk factors [10]. Nonetheless, the performance of FRAX in terms of fracture discrimination is unsatisfactory [11–13].

In the past decade, advanced genome-wide association studies (GWAS) have identified millions of common genetic variants associated with either fracture or fracture-related traits [14–16]. As a highly heritable (50–85%) skeletal measure [3] that predicts fracture risk, bone mineral density (BMD) has been comprehensively investigated in several GWASs, with many common genetic variants been discovered [14–16]. Moreover, extensive cohort resources have enabled the genetic prediction of such heritable clinical risk factors from genotypes through polygenic scores (PGS), which capture information from many single nucleotide polymorphisms (SNPs) assayed from genome-wide genotyping. We previously developed and validated two genome-wide PGSs related to the femoral neck (*PGS*_*FNBMD*_*ldpred*_) and total body BMD (*PGS*_*TBBMD*_*ldpred*_) in the UK Biobank (UKB) cohort [17]. Both genome-wide PGS was proven to be significantly associated with incident fracture risk, even after accounting for FRAX clinical risk factors [17] However, the UKB participants were generally younger and healthier than the general population. Our prior findings thus lack generalizability outside of the UKB cohort. Also, since not all 12 clinical risk factors in FRAX were available in the UKB, a comprehensive evaluation of PGS with complete adjustment was not conducted.

The objective of this study was twofold: firstly, to conduct a comprehensive validation of the predictive power of two previously established genome-wide PGSs in an external cohort and, secondly, to develop a PGS for BMD by combining the information of both *PGS*_*FNBMD*_*ldpred*_ and *PGS*_*TBBMD*_*ldpred*_ using a meta-analytic strategy. The study aimed to assess the PGSs’ ability to stratify fracture risk and to determine if combining PGSs with FRAX would enhance the accuracy of identifying individuals at high risk of osteoporotic fracture.

## Methods

### Study cohort

The Women’s Health Initiative (WHI) study is a nationwide health study aimed at preventing heart disease, breast cancer, and osteoporotic fractures in postmenopausal women [18]. In this study, we used genotype and phenotype data of four WHI sub-studies (the WHI Genomics and Randomized Trials Network (GARNET), the National Heart Lung and Blood Institute (NHLBI), the Population Architecture using Genomics and Epidemiology (PAGE), and the Women’s Health Initiative Memory Study (WHIMS)) acquired through the Database of Genotype and Phenotype (dbGap). As the genetic models were primarily developed and trained using samples of European ancestry, we limited this study to only include Caucasian women to ensure a relatively homogeneous ancestry. Participants who were taking medication that affected BMD (n = 0) and participants who did not have complete information regarding FRAX risk factors were excluded from this study (n = 797). Overall, the data analysis included 9,203 eligible women.

### Ethics approval

This study was approved by the Database of Genotype and Phenotype (dbGap) (https://www.ncbi.nlm.nih.gov/projects/gap/cgi-bin/study.cgi?studyid=phs000200.v12.p3) and the institutional review board at the University of Nevada, Las Vegas. The data was fully anonymized before we accessed them, and UNLV IRB waived the informed consent.

### Fracture and outcome ascertainment

The primary outcome of this study was MOF, which were defined as a composite of hip, humerus, forearm, and clinical vertebral fractures in accordance with the FRAX definition. The follow-up time for WHI participants was calculated from the date of their baseline visit until the occurrence of the first fracture or until the subject’s death. Self-reported fractures were identified by questionnaires. People who did not experience a fracture or death were followed up until the end of the WHI main study, was 12 years after enrollment.

### Clinical risk factors ascertainment

Information regarding age, race, exercise, smoking status, alcohol intake, previous fragility fractures, familial history of fracture, frequency of falls, medication use were collected at baseline using a standard medical questionnaire. Heavy drinking was defined as consume more than three alcoholic drinks per day. Smoking status was categorized following the American Heart Association (AHA) guidelines as “never”, “past”, and “current”. Height was measured in centimeters in standing position, and weight was measured in kilograms using a balance beam.

### Genotype imputation

The genotype data used in this study were obtained from blood samples and acquired through dbGap. Genotyping was performed using either the Illumina (Illumina, San Diego, California) or Affymetrix 6.0 Array Set Platform (Affymetrix, Santa Clara, California). Genotype imputation was carried out using the Sanger Imputation Server, employing the Haplotype Reference Consortium (HRC) reference panel and the Positional Burrows-Wheeler Transform (PBWT) imputation algorithm.

### Polygenic scores

*PGS*_*FNBMD*_*ldpred*_ and *PGS*_*TBBMD*_*ldpred*_ were quantified using LDpred2 with optimal hyperparameters determined previously [17]. For femoral neck BMD and total body BMD, *ρ* thresholds of 0.03 and 0.13, respectively, were used to derive the genome-wide PGSs for each individual in the WHI cohort. Since the PGSs were BMD-related, greater PGS is projected to be associated with higher BMD and lower fracture risk.

To construct the metaPGS for BMD, we used the existing *PGS*_*FNBMD*_*ldpred*_ and *PGS*_*TBBMD*_*ldpred*_ scores. The metaPGS was calculated as follows:

$${PGS}_{i}^{meta}=\frac{\beta_{1}Z_{i1}+\beta_{2}Z_{i2}}{\sqrt{\beta_{1}^{2}+\beta_{2}^{2}+2\beta_{1}\beta_{2}\rho_{1,2}}}$$

where *Z*_*i*1_,*Z*_*i*2_, are the standardized *PGS*_*FNBMD*_*ldpred*_ and *PGS*_*TBBMD*_*ldpred*_ for the *i*th individual, respectively. *β*_1_ and *β*_2_ are the univariate log odds ratios for each score (estimated using logistic regressions), and *ρ*_1,2_ is the Pearson correlation between the *i*th and *j*th scores.

### Statistical analysis

The *PGS*_*FNBMD*_*ldpred*_ and *PGS*_*TBBMD*_*ldpred*_ were standardized with a mean of zero and a standard deviation (SD) of one to enable a standardized comparison of effects. The study categorized participants into three groups based on the percentile distribution (≤10%, 10–90%, and >90%) of each PGS to show the cumulative fracture incidence in individuals with distinct genetic profiles. The observed 10-year incidence of MOF by PGS groups was calculated using the cumulative incidence function (CIF), accounting for the competing mortality risk.

To investigate whether adding PGS would improve the predictive ability of FRAX, we formulated four models: (1) Base model: FRAX risk factors; (2) Base model + *PGS*_*FNBMD*_*ldpred*_; (3) Base model + *PGS*_*TBBMD*_*ldpred*_; (4) Base model + metaPGS. The magnitude of the association between each PGS and MOF/HF risk was assessed using hazard ratios (HRs) and corresponding 95% confidence intervals estimated from the Cox Proportional Hazard Models. Model comparison was performed using the likelihood ratio test. The Cox proportional hazard model’s proportionality assumption was visually inspected and examined using the Schoenfeld residual test [19] The linearity assumption was checked using the martingale residual test [20]. All three PGSs (*PGS*_*FNBMD*_*ldpred*_, *PGS*_*TBBMD*_*ldpred*_, and metaPGS) were evaluated both as continuous and categorical variables in the survival analyses.

The performance of four models in identifying individuals at risk of sustaining a fracture was evaluated using the Area Under the Curve (AUC) and tested for statistical significance using the DeLong test [21]. The Net Reclassification Improvement (NRI) was used to assess the reclassification ability of each model by estimating the predicted risk of fracture for each individual and categorizing them into three risk groups. High risk was defined as a predicted MOF risk ≥ 20% (HF risk ≥ 3%), while low risk was defined as a predicted MOF risk < 20% (HF risk < 3%), based on the National Osteoporosis Foundation’s recommended intervention cutoff [22]. The NRI was used to determine how well the models with PGSs reclassified subjects compared to the base FRAX model. The Integrated discrimination improvement (IDI) was used to measure the direction and extent of the change in the predicted risk. Statistical analyses were conducted in SAS 9.4 (SAS Institute, Inc., Cary, NC, USA).

## Results

### Sample characteristics

The study analyzed a total of 9,203 women who were followed for an average of 12 years. Out of these, 1,255 (13.6%) women sustained at least one MOF during the follow-up period, with 600 (6.5%) experiencing HF. The average duration of follow-up for women who experienced at least one MOF was 6.7 years. The baseline characteristics of the participants were compared between fracture status groups and presented in **Table 1**. Older age, lower body mass index and had higher alcohol consumption were associated with a higher likelihood of fracture. Participants who experienced fractures also had a higher prevalence of prior fractures, family history of HFs, and falls in the past 12 months. The distribution of *PGS*_*FNBMD*_*ldpred*_, *PGS*_*TBBMD*_*ldpred*_, and metaPGS in the WHI cohort was generally normal.

**Table 1:** Characteristics of 9,203 women stratified by Major Osteoporosis Fracture (MOF) and Hip Fracture (HF) status.

	Subjects without fracture (N = 7,948)	Subjects with MOF (N = 1,255)	P value	Subjects with HF (N = 600)	P value
Age (year), mean (SD)	66.96 ± 6.40	69.07 ± 5.74	**<0.01**	70.18 ± 5.00	**<0.01**
Weight (kg), mean (SD)	75.62 ± 15.79	73.24 ± 15.11	**<0.01**	72.07 ± 15.23	**<0.01**
Height (cm), mean (SD)	161.36 ± 5.96	161.40 ± 6.10	0.81	161.76 ± 6.13	0.10
Body mass index (kg/m^2^), mean (SD)	29.00 ± 5.79	28.10 ± 5.59	**<0.01**	27.50 ± 5.42	**<0.01**
Smoking, n (%)					
Never	4,033 (50.74%)	651 (51.87%)	0.70	323 (53.83%)	0.31
Past	3,209 (40.37%)	491 (39.12%)		230 (38.33%)	
Current	706 (8.88%)	113 (9.00%)		47 (7.83%)	
≥ 3 alcohol drinks per day, n (%)					
Yes	119 (1.49%)	22 (1.75%)	0.46	9 (1.50%)	0.99
No	7,829 (98.50%)	1,233 (98.25%)		591 (98.50%)	
Rheumatoid arthritis, n (%)					
Yes	376 (4.73%)	72 (5.74%)	0.14	43 (7.17%)	**0.01**
No	7,572 (95.27%)	1,183 (94.26%)		557 (92.83%)	
Previous fragility fracture, n (%)					
Yes	3,454 (43.46%)	697 (55.54%)	**<0.01**	330 (55.00%)	**<0.001**
No	4,494 (56.54%)	558 (44.46%)		270 (45.00%)	
Familial history of fracture, n (%)					
Yes	1,150 (14.47%)	230 (18.33%)	**<0.01**	119 (19.83%)	**<0.01**
No	6,798 (85.53%)	1025 (81.67%)		481 (80.17%)	
*PGS*_*FNBMD*_*ldpred*_, mean (SD)	0.08 ± 0.80	0.03 ± 0.81	**0.04**	0.04 ± 0.82	0.30
*PGS*_*TBBMD*_*ldpred*_, mean (SD)	-0.62 ± 0.79	-0.68 ± 0.79	**0.02**	-0.69 ± 0.79	0.06
MetaPGS, mean (SD)	0.32 ± 0.81	0.26 ± 0.80	**<0.01**	0.27 ± 0.80	0.12

*Significant results are in boldface.

### Crude 10-year cumulative incidence by PGSs

**Fig 1:**
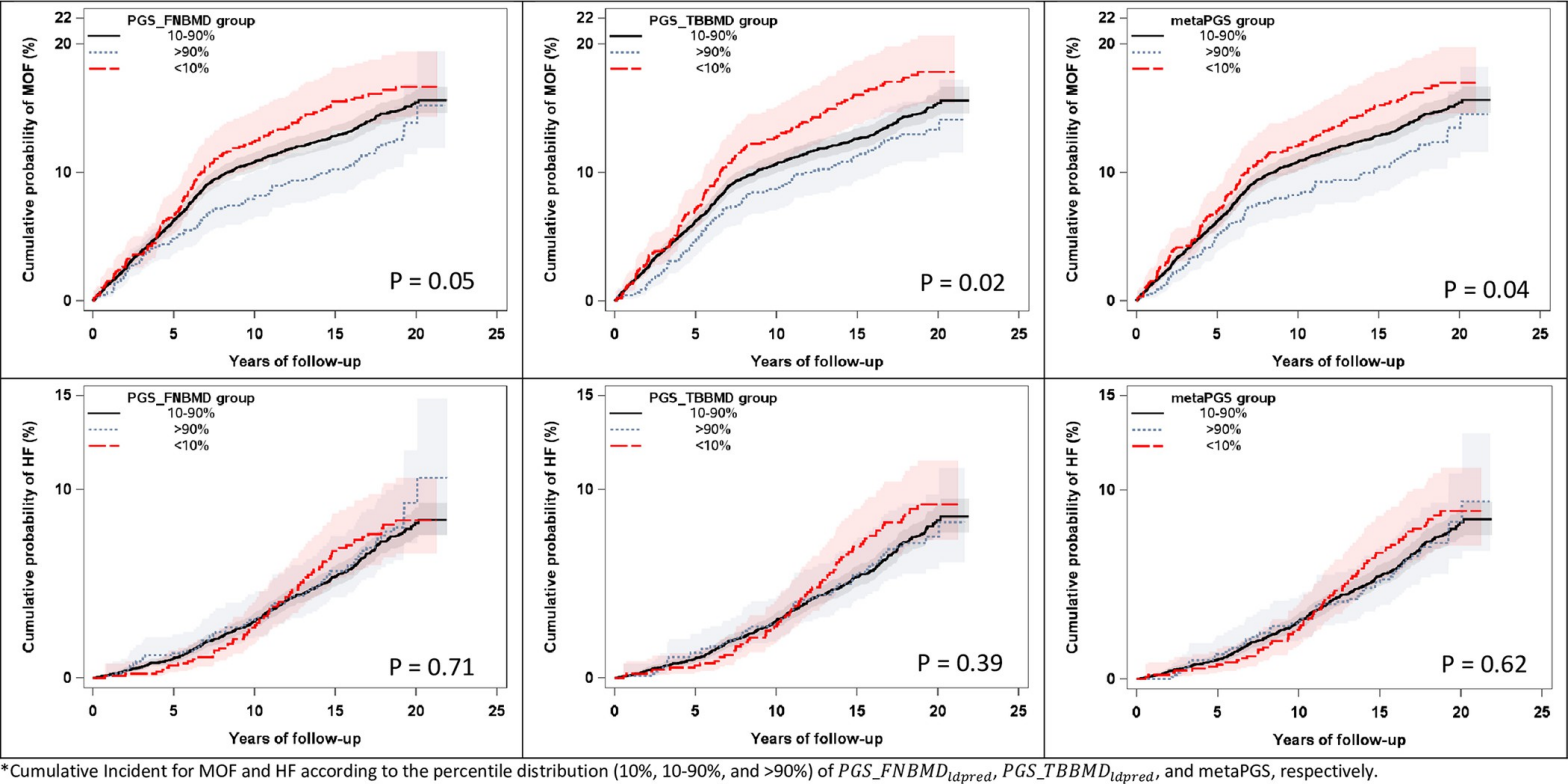
Cumulative incident function plot for fracture according to decile of the genome-wide polygenic score (PGS) in WHI. Shaded Regions Denote 95% Confidence Intervals. *Cumulative Incident for MOF and HF according to the percentile distribution (10%, 10–90%, and >90%) of *PGS*_*FNBMD*_*ldpred*_, *PGS*_*TBBMD*_*ldpred*_, and metaPGS, respectively.

**Fig 1** presents the crude 10-year cumulative incidence of MOF and HF according to three PGS groups. After accounting for competing mortality, there were borderline significant differences observed across the group of *PGS*_*FNBMD*_*ldpred*_ for MOF (p = 0.05), but not for HF (p = 0.71). Similar results were observed across the groups of *PGS*_*TBBMD*_*ldpred*_ (MOF: p = 0.02) and metaPGS (MOF: p = 0.04). Individuals with lower *PGS*_*FNBMD*_*ldpred*_, *PGS*_*TBBMD*_*ldpred*_, and metaPGS had higher incidences of MOF and HF.

### Association between PGSs and fracture

The Cox Proportional Hazard model showed that when treating PGSs as continuous variables, all *PGS*_*FNBMD*_*ldpred*_, *PGS*_*TBBMD*_*ldpred*_, and metaPGS were not significantly associated with MOF or HF after adjusting for FRAX risk factors **(Tables 2 and 3)**. We next used PGS groups and included PGS in Cox regression models as categorical variables. The results were similar. Individuals in the top 10% of metaPGS distribution had no increased MOF risk compared with the bottom 10% of the individuals (HR: 0.84, 95% CI, 0.65–1.10). Compared to the women in the high *PGS*_*FNBMD*_*ldpred*_, *PGS*_*TBBMD*_*ldpred*_, and metaPGS groups, women in the low PGS groups did not show a significantly higher risk of MOF **(Table 4)**. Similar findings with HF outcomes were also observed **(Table 5)**.

**Table 2:** Results of Cox Proportional Hazard regression analyzing the effect of baseline variables on Major Osteoporosis Fracture (MOF) the Women’s Health Initiative (WHI) cohort.

Variable	Model 1: FRAX Base Model HR (95% CI) in MOF	Model 2: FRAX + *PGS*_*FNBMD*_*ldpred*_ HR (95% CI) in MOF	Model 3: FRAX + *PGS*_*TBBMD*_*ldpred*_ HR (95% CI) in MOF	Model 4: FRAX + metaPGS HR (95% CI) in MOF
**Age**	**1.07 (1.06–1.08)**	**1.07 (1.06–1.08)**	**1.07 (1.06–1.08)**	**1.07 (1.06–1.08)**
**Body weight**	**0.99 (0.99–1.00)**	**0.99 (0.99–1.00)**	**0.99 (0.99–1.00)**	**0.99 (0.99–1.00)**
**Height**	**1.02 (1.01–1.03)**	**1.02 (1.01–1.03)**	**1.02 (1.01–1.03)**	**1.02 (1.01–1.03)**
**Previous osteoporotic fracture**	**1.51 (1.34–1.70)**	**1.50 (1.33–1.69)**	**1.51 (1.34–1.70)**	**1.51 (1.34–1.70)**
**Parental history of hip fracture**	**1.22 (1.05–1.42)**	**1.22 (1.05–1.42)**	**1.22 (1.05–1.43)**	**1.22 (1.05–1.42)**
**Rheumatoid arthritis**	1.24 (0.96–1.60)	1.24 (0.96–1.60)	1.24 (0.96–1.60)	1.24 (0.96–1.60)
**Current smoking**	**1.43 (1.15–1.77)**	**1.43 (1.15–1.77)**	**1.42 (1.15–1.77)**	**1.43 (1.15–1.77)**
**Daily drinking > 3**	1.09 (0.69–1.72)	1.09 (0.69–1.72)	1.09 (0.69–1.73)	1.09 (0.69–1.72)
**Secondary osteoporosis**	1.03 (0.90–1.18)	1.03 (0.90–1.18)	1.03 (0.90–1.17)	1.03 (0.90–1.18)
**PGS**	NA	0.96 (0.90–1.04)	1.02 (0.95–1.10)	1.01 (0.94–1.09)

*Significant results are in boldface.

**Table 3:** Results of Cox Proportional Hazard regression analyzing the effect of baseline variables on Hip Fracture (HF) in the Women’s Health Initiative (WHI) cohort.

Variable	Model 1: FRAX Base Model HR (95% CI) in HF	Model 2: FRAX + *PGS*_*FNBMD*_*ldpred*_ HR (95% CI) in HF	Model 3: FRAX + *PGS*_*TBBMD*_*ldpred*_ HR (95% CI) in HF	Model 4: FRAX + *metaPGS* HR (95% CI) in HF
**Age**	**1.12 (1.11–1.14)**	**1.12 (1.11–1.14)**	**1.13 (1.11–1.15)**	**1.13 (1.11–1.14)**
**Body weight**	**0.99 (0.98–1.00)**	**0.99 (0.98–1.00)**	**0.99 (0.98–1.00)**	**0.99 (0.98–1.00)**
**Height**	**1.04 (1.02–1.05)**	**1.04 (1.02–1.05)**	**1.04 (1.02–1.05)**	**1.04 (1.02–1.05)**
**Previous osteoporotic fracture**	**1.45 (1.22–1.71)**	**1.45 (1.22–1.71)**	**1.45 (1.22–1.72)**	**1.45 (1.22–1.71)**
**Parental history of hip fracture**	**1.30 (1.06–1.60)**	**1.30 (1.06–1.60)**	**1.31 (1.06–1.61)**	**1.30 (1.06–1.61)**
**Rheumatoid arthritis**	**1.72 (1.25–2.38)**	**1.72 (1.25–2.38)**	**1.72 (1.25–2.38)**	**1.72 (1.25–2.38)**
**Current smoking**	**1.56 (1.13–2.15)**	**1.56 (1.13–2.15)**	**1.55 (1.13–2.14)**	**1.56 (1.13–2.14)**
**Daily drinking > 3**	0.85 (0.42–1.70)	0.85 (0.42–1.71)	0.85 (0.42–1.72)	0.85 (0.42–1.71)
**Secondary osteoporosis**	1.13 (0.94–1.36)	1.13 (0.94–1.36)	1.12 (0.93–1.35)	1.13 (0.93–1.35)
**PGS**	NA	1.01 (0.91–1.12)	1.09 (0.98–1.21)	0.96 (0.86–1.06)

*Significant results are in boldface.

**Table 4:** Hazard ratio for Major Osteoporotic Fractures (MOF) in low vs. high PGS groups: Results of Cox Proportional Hazard Models adjusted for FRAX clinical risk factors.

Variable	Model 1: FRAX Base Model HR (95% CI) in MOF	Model 2: FRAX + *PGS*_*FNBMD*_*ldpred*_ HR (95% CI) in MOF	Model 3: FRAX + *PGS*_*TBBMD*_*ldpred*_ HR (95% CI) in MOF	Model 4: FRAX + metaPGS HR (95% CI) in MOF
**Age**	**1.07 (1.06–1.08)**	**1.07 (1.06–1.08)**	**1.07 (1.06–1.08)**	**1.07 (1.05–1.08)**
**Body weight**	**0.99 (0.99–1.00)**	**0.99 (0.99–1.00)**	**0.99 (0.99–1.00)**	**0.99 (0.99–1.00)**
**Height**	**1.02 (1.01–1.03)**	**1.02 (1.01–1.03)**	**1.01 (1.00–1.03)**	**1.02 (1.01–1.03)**
**Previous osteoporotic fracture**	**1.51 (1.34–1.70)**	**1.50 (1.33–1.69)**	**1.52 (1.35–1.71)**	**1.50 (1.33–1.69)**
**Parental history of hip fracture**	**1.22 (1.05–1.42)**	**1.22 (1.05–1.42)**	**1.22 (1.05–1.42)**	**1.22 (1.05–1.42)**
**Rheumatoid arthritis**	1.24 (0.96–1.60)	1.24 (0.96–1.60)	1.24 (0.96–1.60)	1.24 (0.96–1.60)
**Current smoking**	**1.43 (1.15–1.77)**	**1.43 (1.15–1.77)**	**1.40 (1.13–1.74)**	**1.43 (1.15–1.78)**
**Daily drinking > 3**	1.09 (0.69–1.72)	1.09 (0.69–1.72)	1.10 (0.69–1.73)	1.09 (0.69–1.71)
**Secondary osteoporosis**	1.03 (0.90–1.18)	1.03 (0.90–1.18)	1.04 (0.91–1.18)	1.03 (0.90–1.18)
**PGS**				
**<10%**	NA	Ref.	Ref.	Ref.
**10–90%**	NA	0.92 (0.77–1.11)	0.88 (0.74–1.04)	0.93 (0.78–1.12)
**>90%**	NA	0.84 (0.64–1.09)	0.85 (0.66–1.10)	0.84 (0.65–1.10)

*Significant results are in boldface.

**Table 5:** Hazard ratio for Hip Fractures (HF) in low vs. high PGS groups: Results of Cox Proportional Hazard Models adjusted for FRAX clinical risk factors.

Variable	Model 1: FRAX Base Model HR (95% CI) in HF	Model 2: FRAX + *PGS*_*FNBMD*_*ldpred*_ HR (95% CI) in HF	Model 3: FRAX + *PGS*_*TBBMD*_*ldpred*_ HR (95% CI) in HF	Model 4: FRAX + *metaPGS* HR (95% CI) in HF
**Age**	**1.12 (1.11–1.14)**	**1.12 (1.11–1.14)**	**1.12 (1.11–1.14)**	**1.12 (1.11–1.14)**
**Body weight**	**0.99 (0.98–1.00)**	**0.99 (0.98–1.00)**	**0.99 (0.98–1.00)**	**0.99 (0.98–1.00)**
**Height**	**1.04 (1.02–1.05)**	**1.04 (1.02–1.05)**	**1.04 (1.02–1.05)**	**1.04 (1.02–1.05)**
**Previous osteoporotic fracture**	**1.45 (1.22–1.71)**	**1.45 (1.23–1.72)**	**1.45 (1.22–1.71)**	**1.45 (1.22–1.71)**
**Parental history of hip fracture**	**1.30 (1.06–1.60)**	**1.30 (1.06–1.61)**	**1.30 (1.05–1.60)**	**1.30 (1.06–1.60)**
**Rheumatoid arthritis**	**1.72 (1.25–2.38)**	**1.72 (1.25–2.38)**	**1.72 (1.25–2.38)**	**1.72 (1.25–2.38)**
**Current smoking**	**1.56 (1.13–2.15)**	**1.56 (1.14–2.15)**	**1.56 (1.13–2.14)**	**1.56 (1.13–2.15)**
**Daily drinking > 3**	0.85 (0.42–1.70)	0.86 (0.42–1.72)	0.85 (0.42–1.70)	0.85 (0.42–1.71)
**Secondary osteoporosis**	1.13 (0.94–1.36)	1.13 (0.94–1.36)	1.13 (0.94–1.36)	1.13 (0.94–1.36)
**PGS**				
**<10%**	NA	Ref.	Ref.	Ref.
**10–90%**	NA	1.02 (0.78–1.33)	0.99 (0.77–1.27)	1.02 (0.79–1.31)
**>90%**	NA	1.23 (0.86–1.75)	1.07 (0.74–1.54)	1.12 (0.78–1.60)

*Significant results are in boldface.

### Model evaluation

To evaluate the ability of *PGS*_*FNBMD*_*ldpred*_, *PGS*_*TBBMD*_*ldpred*_, and metaPGS to discriminate fractures over FRAX, the study used the concordance index (C-index), as shown in **Table 6**. The FRAX base model showed moderate discrimination of MOF and HF, with C-index values of 0.623 (95% CI, 0.609 to 0.641) and 0.702 (95% CI, 0.609 to 0.718), respectively. However, no improvement was observed in discriminating MOF and HF when PGSs were added to the base FRAX model.

**Table 6:** Concordance index (and 95% confidence interval) of predicted and observed fracture risk for the model with and without PGS.

	Model 1: FRAX Base Model HR (95% CI)	Model 2: FRAX + *PGS*_*FNBMD*_*ldpred*_ HR (95% CI)		Model 3: FRAX + *PGS*_*TBBMD*_*ldpred*_ HR (95% CI)		Model 4: FRAX + *metaPGS* HR (95% CI)	
	C-index	C-index	p-value	C-index	p-value	C-index	p-value
MOF	0.623 (0.609–0.641)	0.623 (0.608–0.641)	0.72	0.623 (0.609–0.641)	0.60	0.623 (0.602–0.641)	0.92
Hip fracture	0.701 (0.609–0.718)	0.702 (0.609–0.718)	0.98	0.702 (0.609–0.723)	0.33	0.702 (0.611–0.785)	0.63

In the reclassification analysis, compared to the FRAX base model, models with *PGS*_*FNBMD*_*ldpred*_ (0.37%, p = 0.33), *PGS*_*TBBMD*_*ldpred*_ (0.5%, p = 0.14), or metaPGS (0.05%, p = 0.99) did not improve the reclassification of MOF (**Table 7**). The model incorporated *PGS*_*FNBMD*_*ldpred*_ correctly reclassified five individuals (0.45%) to the high-risk group, and eighteen (0.27%) individuals who did not experience a MOF were correctly reclassified to the low-risk group. For the model including metaPGS, two (0.18%) individuals were correctly reclassified to the high-risk group, and seven (0.11%) women who did not experience a MOF were correctly reclassified to the low-risk group. The continuous NRI revealed that the improvement in MOF reclassification contributed by *PGS*_*FNBMD*_*ldpred*_, *PGS*_*TBBMD*_*ldpred*_, and metaPGS overall were 0.63% (p = 0.87), 0.88% (p = 0.81), and 1.54% (p = 0.68), respectively. Better reclassification provided by PGSs was observed in HF prediction. *PGS*_*FNBMD*_*ldpred*_ and metaPGS improved the reclassification of HF significantly by 1.2% (p = 0.04) and 1.7% (p = 0.05). In the context of HF prediction, the FRAX+metaPGS model correctly reclassified 6 out of 600 (1.1%) participants with HF upward to the high-risk group, and 58 out of 8,603 (0.8%) women who did not experience HF were correctly reclassified downward to the low-risk group. Additionally, the inclusion of *PGS*_*TBBMD*_*ldpred*_ to the base FRAX model let to a significant increase in IDI of 1.93% (p = 0.01) for predicting HF. However, the overall improvement in fracture event reclassification provided by the PGSs models was minimal.

**Table 7:** Reclassification table of 10-year MOF and HF stratified by event status.

Reclassification										
	Non-fracture group		Fracture group		NRI (category)	*p*	NRI (continuous)	*p*	IDI	*p*
	**Reclassification down**	**Reclassification up**	**Reclassification up**	**Reclassification down**						
***PGS*_*FNBMD***_***ldpred***_										
**MOF**	18 (0.27%)	19 (0.29%)	5 (0.45%)	7 (0.64%)	0.37%	0.33	0.63%	0.87	0.49%	0.08
**HF**	13 (0.18%)	25 (0.35%)	1 (0.18%)	0 (0%)	-0.17%	0.42	-1.11%	0.87	0.10%	0.76
***PGS*_*TBBMD***_***ldpred***_										
**MOF**	9 (0.14%)	5 (0.1%)	6 (0.54%)	4 (0.36%)	0.5%	0.14	0.88%	0.81	0.14%	0.69
**HF**	102 (1.42%)	113 (1.57%)	8 (1.45%)	6 (1.09%)	1.2%	**0.04**	6.25%	0.36	1.93%	**0.01**
***metaPGS***										
**MOF**	7 (0.11%)	9 (0.14%)	2 (0.18%)	3 (0.27%)	0.05%	0.99	1.54%	0.68	0.06%	0.46
**HF**	58 (0.8%)	62 (0.8%)	6 (1.1%)	3 (0.5%)	1.7%	**0.05**	1.48%	0.83	0.76%	0.06

*Significant results are in boldface. NRI = net reclassification improvement; IDI = integrated discriminative improvement.

## Discussion

Implementing individual-level genome-wide PGSs summarizing the underlying genetic predisposition of certain diseases in the clinical setting holds excellent promise. Previously, we developed and internally validated two genome-wide BMD-related PGSs using data from the UKB cohort [17]. Our findings indicated that both PGSs were significantly associated with incident fractures, even after being adjusted for FRAX clinical risk factors [17]. In the current study, we replicated the two BMD-associated PGSs from previous work [17] and additionally derived a third PGS—metaPGS combining the effect of the two established PGSs and evaluated their predictive effect in an independent WHI postmenopausal women sample. We examined whether the PGSs could stratify individuals into different risk strata within this external validation cohort and the predictive ability of each PGS beyond the FRAX tool.

Our findings showed that both the BMD-related genome-wide PGSs and the metaPGS did not perform as well and were not significantly associated with fractures in the WHI cohort. Moreover, adding genetic information to the FRAX tool was associated with minimal improvements in predicted probabilities of HF among elderly Caucasian women. These findings were in discordance with our previous research findings [17], of which PGSs calculated based on genome-wide significant loci showed significant association with fractures and provided minor improvement of fracture prediction beyond the base model consisting of convention clinical risk factors. Previous studies have produced mixed results regarding the effectiveness of polygenic scores in improving fracture prediction accuracy. For example, Thao et al. reported that genetic profiling of 63 BMD-related genetic variants could enhance fracture prediction performance when compared to the Garvan fracture risk calculator [23]. Our prior work demonstrated that incorporating genetic information from 81 BMD-related genetic variants could improve fracture prediction performance beyond FRAX [24]. A more recent study generated and validated a genome-wide PGS for speed of sound (SOS) also reported a consistent association with fracture risk [25]. However, Eriksson and colleagues reported only minor improvements in fracture prediction when adding a PGS to a base model consisting of age, height, and weight [26].

The performance of PGS in different cohorts is affected by many factors. The PGSs may have been overfitted to the training sample, meaning that it is too closely tied to the specific individuals and variants used in the training sample. This inconsistency can result in poor performance when applied to a validation sample. Compared to the UK Biobank genotype data, WHI genotyping data has fewer SNPs and less genome coverage. The hyper-parameters tuned in UKB might not be as optimal in WHI due to heterogeneity between the training and testing cohorts. Also, the allele frequencies of SNPs will vary by population, together with the causal variants and their effect sizes [27, 28]. Moreover, genotyping imputation is one way to introduce variability in calculated PGSs at the individual level without changing the underlying genetic model [29]. The UKB carried out imputation on the genotype data using SHAPEIT3 and IMPUTE4, whereas the WHI was imputed using Positional Burrows-Wheeler Transform (PBWT) imputation algorithm. Lastly, while genetics plays a role in determining traits and conditions, other factors such as the environment, lifestyle, and sociodemographic characteristics can also influence the expression of these traits. So, even individuals with similar ancestry may have different risks for certain conditions based on these other factors, affecting the accuracy of genetic risk predictions [30].

This study comprehensively validated the predictive power of two previously established genome-wide PGSs, as well as a metaPGS that combined information from these two PGSs. Additionally, we assessed the ability of PGSs to stratify fracture risk and to determine if combining PGSs with FRAX would enhance the accuracy of identifying individuals at high risk of osteoporotic fracture. It is essential to acknowledge the limitations of this study. First, the sample size of current study is relatively small to replicate findings discovered in a cohort of half million (UKB); Second, fracture risk of WHI may not be fully captured by the PGS calculated using the hyper-parameters derived in other cohorts. Finally, our study only included individuals of European ancestry, which may limit the generalizability of our findings.

Early detection of high-risk individuals could lead to efficacious preventive and therapeutic interventions. However, based on the hyper-parameters derived in the UKB, we could not replicate our prior findings in this external independent WHI cohort. The two BMD-related genome-wide PGSs and the metaPGS were not significantly associated with fractures in the WHI cohort. Adding genetic information to the FRAX tool was associated with minimal improvements in predicted HF probabilities among elderly Caucasian women.
